# Quality of Life after Treatment for Pelvic Organ Prolapse in Real-World Study: Recommendations, Vaginal Pessary, and Surgery

**DOI:** 10.3390/medicina60040547

**Published:** 2024-03-28

**Authors:** Dominyka Mančinskienė, Miglė Mikėnaitė, Mark Barakat, Justina Kačerauskienė, Dalia Regina Railaitė, Laima Maleckienė, Arnoldas Bartusevičius, Eglė Bartusevičienė

**Affiliations:** 1 Department of Obstetrics and Gynaecology, Lithuanian University of Health Sciences, LT-44307 Kaunas, Lithuania; justina.kacerauskiene@lsmu.lt (J.K.); daliaregina.railaite@lsmu.lt (D.R.R.); laima_maleckiene@yahoo.com (L.M.); arnoldas.bartusevicius@lsmu.lt (A.B.); egle.bartuseviciene@lsmu.lt (E.B.); 2 Faculty of Medicine, Lithuanian University of Health Sciences, LT-44307 Kaunas, Lithuania; mikenaitemigle@gmail.com (M.M.); barakat.markas@gmail.com (M.B.)

**Keywords:** pelvic organ prolapse, pessary, surgery, recommendations, global improvement, quality of life

## Abstract

*Background and Objectives:* Pelvic organ prolapse (POP) is a common condition in women, with its prevalence increasing with age, and can significantly impact the quality of life (QOL) of many individuals. The objective of this study was to assess the overall improvement, quality of life, and continuation of primary treatment for POP over a 24-month period in a real-world setting. *Materials and Methods:* This is a prospective, observational, follow-up study of women with symptomatic POP who, as a primary treatment, opted for recommendations (lifestyle changes and pelvic floor muscle training), pessary therapy, or surgery. The primary outcome measure was a subjective improvement at the 24-month follow-up, measured with the Patient Global Impression of Improvement (PGI-I) scale. Secondary outcome measures were the continuation rate of the primary treatment method, reason for discontinuation, and the quality of life evaluated with the P-QoL questionnaire. *Results:* We included 137 women, with 45 women (32.8%) in the recommendations group, 39 (28.5%) in the pessary group, and 53 women (38.7%) in the surgery group. After 24 months, surgery, in comparison with pessary treatment and recommendations, resulted in significantly more women reporting a subjective improvement: 89.6%, 66.7%, and 22.9% (*p* < 0.001), respectively. Overall, 52% of women from the recommendations group and 36.4% from the pessary group switched to another treatment or discontinued the primary treatment within 24 months. However, women who continued the primary treatment, pessary use, and surgery showed similar subjective improvements (90.5% and 89.6%, respectively) and quality-of-life improvement. *Conclusions:* The chance of significant improvement was higher following surgery. However, after 24 months, both vaginal pessaries and surgery showed an important quality-of life improvement and can be proposed as primary treatment methods for pelvic organ prolapse.

## 1. Introduction

Pelvic organ prolapse (POP) is a common condition in women and its prevalence increases with age [1]. About 50% of all women eventually acquire POP, which may not necessarily demonstrate symptoms but could only involve anatomical shifting [2]. However, this condition can substantially and negatively impact the quality of life (QOL) of many women [3,4]. Treatment options are limited with symptomatic POP and can include conservative management (pelvic floor muscle training (PFMT), lifestyle changes, and vaginal pessaries) or prolapse surgery [5,6]. The number of patients undergoing surgery for POP is expected to increase by up to 13% [7]. Prolapse surgery reduces pelvic floor symptoms by restoring the anatomy but is associated with surgical complications, and around 20% of woman experience a recurrence of bothersome POP symptoms [8]. It is believed that conservative approaches to the treatment of prolapse are reserved for women with mild or moderate prolapse, those who wish to have more children, the frail, and those unwilling to undergo surgery or the contrary—those awaiting surgery [5,9,10]. However, in some studies, the use of a vaginal pessary allowed the avoidance of surgery in 72% of women with POP, and pelvic floor muscle training resulted in a great subjective improvement in prolapse symptoms [10,11]. The increasing number of studies evaluating vaginal pessaries’ impact on POP shows that both pessary use and surgery can improve the condition and prolapse symptoms of women [12]. Although pessary therapy is considered conservative, studies have reported that adverse effects such as pessary expulsion, discomfort, pain, and excessive discharge are associated with a discontinuation of treatment in 24–49% of women within 12 to 24 months [3,13,14,15].

The choice of treatment depends on various factors, including perceptions of efficacy and risk associated with the treatment method, women’s characteristics and clinical profile, preferences of both women and gynecologists, and variations in healthcare systems. Studies comparing conservative treatment with surgery for symptomatic POP primarily focus on effectiveness, symptom relief, discontinuation rates, and side effects while addressing quality of life to a lesser extent. The objective of this cohort study was to investigate the overall improvement in complaints, quality of life, and continuation of primary treatment over a 24-month period in a real-world setting.

## 2. Materials and Methods

This study to validate the Lithuanian version of the Prolapse Quality-of-Life (P-QoL) questionnaire among Lithuanian-speaking women with urogenital prolapse was carried out from 1 January 2020 to 31 December 2020 at the tertiary referral Department of the Obstetrics and Gynaecology of the Lithuanian University of Health Sciences. Alongside the P-QoL validation process, a prospective, observational study among women with symptomatic urogenital prolapse was set up. The approval of the Regional Biomedical Research Ethics Committee was received for this study (No. BE-2-79). The inclusion criteria were as follows: symptomatic POP (complaints of vaginal bulge or the feeling of something coming down or out of the vagina), women older than 18 years, and native Lithuanian speakers. As for the exclusion criteria, these were as follows: current pregnancy, ongoing treatment for an oncological disease, previous surgery for the correction of POP or urinary incontinence, previous treatment with a pessary, and any neurological or mental illness preventing the completion of the questionnaire. Coexisting urinary incontinence or bowel symptoms were not among the exclusion criteria.

The eligible women with symptomatic POP who met the inclusion criteria were methodically evaluated and counselled about the POP by one of three urogynaecologists (E.B., J.K., and D.R.R.). The treatment options, including recommendations for lifestyle changes and pelvic floor muscle training (PFMT), pessary treatment, and surgery were explained in a non-directive manner. When a woman opted for one of these treatments, she was invited to participate in the study. Written informed consent was obtained from all participants before they entered into the study. 

All women received written recommendations about lifestyle changes (weight loss, smoking cessation, avoiding constipation, heavy lifting, high-impact exercise, and bladder irritants such as caffeine) and pelvic floor muscle training and were advised to follow them. For postmenopausal women, local estrogen therapy was recommended as well. 

If a woman opted for pessary treatment, supportive or occlusive pessaries could be used according to the judgement of the gynecologist. All three urogynecologists were skilled in pessary fitting. In cases of pessary expulsion, a trial of another size or shape of pessary was tested. The pessary fitting was considered successful if the woman felt comfortable with the pessary and there was no pessary expulsion. After placement, all women received instructions about the pessary treatment. During this study, only ring pessaries (with or without a central support) were placed. There was no case among the study participants where a pessary was opted for and placed due to contraindications for surgery. All women performed self-management; thus, the frequency of cleaning was left to their personal judgement, but this was no less frequently than every 3 months. A follow-up visit 6–8 weeks after placement was planned. If the woman was satisfied with the pessary, routine visits to the gynecologist (once per year) were advised. Women were encouraged to return earlier if they had any complaints or if they lost the pessary. 

The surgical intervention involved correcting the compartments requiring surgery, as determined at the urogynecologist’s discretion. Anterior or posterior colporrhaphy was considered the standard procedure for repairing anterior or posterior vaginal wall prolapse, respectively. For uterine prolapse, the Manchester procedure, vaginal hysterectomy, and colpocleisis were performed. 

All women were asked to self-complete the validated Lithuanian version of the P-QoL questionnaire, which contains 20 questions representing nine quality-of-life domains: the general health of the woman, prolapse impact, daily role limitations, physical and social limitations, the impact on personal relationships and emotions, the effect on sleep/energy, and the severity measures. The scores in each domain ranged from 0 to 100. Higher scores indicated a greater quality-of-life impairment, while a low total score indicated good quality of life. Additionally, there were 18 questions related to urinary, bowel, and vaginal symptoms, which did not have an assigned score. Responses included “not applicable”, “none”, “a little”, “moderately”, and “a lot”, except for the first question, which ranged between 1 and 5 on the Likert scale. 

Demographic and clinical data included patients’ age, menopausal status, body mass index (BMI), parity, mode of delivery, risk factors for POP, and stage of prolapse using the Pelvic Organ Prolapse Quantification System (POP-Q).

A follow-up at 24 months was planned as part of the study. However, due to the restrictions during the COVID-19 pandemic, we faced difficulties and reluctance from the women to come for a follow-up visit. It was decided to contact the study participants by phone and fill out the questionnaires during an interview in December 2022.

The primary outcome measure was a subjective improvement at the 24-month follow-up, measured with the Patient Global Impression of Improvement (PGI-I) scale. The simple and easy-to-use PGI-I scale is a method used to evaluate the success of treatment for symptomatic POP. It contains a single question that aids in assessing the current condition compared to before treatment, using a Likert scale ranging from 1 (very much better) to 7 (very much worse). We considered values 1 to 2 (very much better or much better) as a subjective improvement at the 24-month follow-up. The secondary outcome measures included the continuation rate of the primary treatment method, reasons for discontinuation (such as preference for prolapse surgery, complaints of excessive discharge, pessary expulsion, sexual problems, or other), and the quality of life of women evaluated at the 24-month follow-up using the P-QoL questionnaire.

The sample size was chosen pragmatically, including all women with symptomatic POP from the P-QoL questionnaire validation study. Statistical analysis was carried out by using the SPSS (Statistical Package for the Social Sciences), version 29.0 for Windows (Chicago, IL, USA). Categorical data are presented as numbers and percentages, while the ordinal and continuous data are presented as medians and interquartile ranges (IQRs). We sorted the descriptive statistics, descriptive demographics, and clinical characteristics with a non-parametric chi-squared test. This method was applied for the PGI-I success rate as well. The Kruskal–Wallis test was used for the difference between age, body mass index (BMI), and parity. A calculation was made of the baseline and follow-up median scores and IQRs for each P-QoL questionnaire domain, and the differences between the treatment groups were evaluated by the non-parametric Kruskal–Wallis test. Statistical significance was set at *p* < 0.05. 

## 3. Results

**Figure 1:**
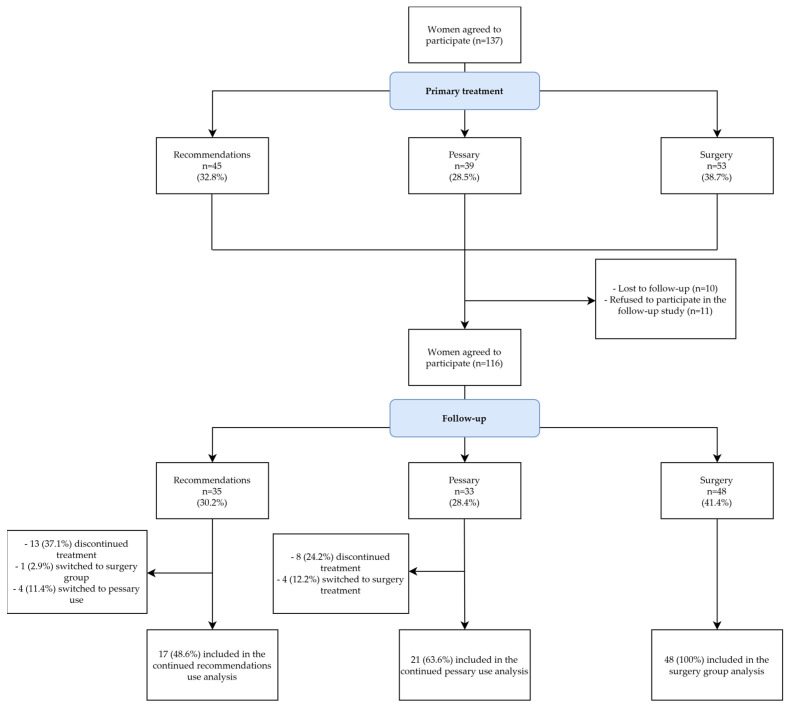
Patient flow chart of the clinical follow-up study.

We approached a total of 137 women with symptomatic POP. Overall, 116 women agreed to answer questionnaires during the phone interview (Figure 1). Data on the primary outcomes were available for 35 women in the recommendations group, for 33 women in the pessary group, and for 48 women in the surgery group. The median follow-up time was 24 months (IQR 19–31). 

The baseline demographic and clinical characteristics of all participants are presented in Table 1. Women in the surgery group were significantly older. Significantly more women in the surgery and pessary groups were postmenopausal and had vaginal atrophy. With regard to the POP-Q stage, the majority of women were stage 3 in the surgery group (88.7%) and the pessary group (82.1%), while the majority of those in the recommendations group were stage 2 (55.6%). The three groups were not significantly different with respect to BMI, smoking, constipation, parity, number of vaginal deliveries, and cesarean section. 

The primary outcomes are presented in Table 2. With respect to the primary treatment methods, at 24 months, a subjective improvement was reported by 89.6% of women in the surgery group, by 66.7% of women in the pessary group, and only by 22.9% of women in the recommendations group (*p* < 0.001). Regarding the study participants who continued the primary treatment method, the proportion of women reporting an improvement increased in the pessary group, which was similar to that in the surgery group: 90.5% and 89.6%, respectively. Although the proportion of women who reported an improvement with the continued use of the recommendations also increased and was 35.3%, the treatment with this method was significantly less successful compared to the continued vaginal pessary use and surgery groups.

The continuation rate at 24 months was 63.6% (21 of 33 women) in the pessary group and 48% (17 of 35 women) in the recommendations group (Figure 1). The reasons for discontinuation or switching to another treatment method are presented in Table 3. Overall, 13 of 35 (37.1%) women in the recommendations group and 8 of 33 (24.2%) women in the pessary group discontinued the initial treatment and did not opt for any other method of POP treatment. 

The findings regarding the impact of prolapse on the quality of life of women are presented in Table 4. The P-QoL domain scores at baseline indicated that women in the surgery group had a greater quality-of-life impairment with respect to general health perception, daily role limitations, the impact on emotions, and the POP severity measures (*p* < 0.05). Meanwhile, the P-QoL scores at 24 months were significantly higher in the recommendations group in most domains, except for the social limitations and personal relationships domains. In addition, there was no significant difference in all P-QoL domain scores between the pessary and surgery groups at the 24-month follow-up, indicating that quality of life using a pessary and after surgery was similar.

## 4. Discussion

### 4.1. Main Findings

This real-world study demonstrated that surgery, compared to pessary treatment and recommendations for lifestyle changes and pelvic floor muscle training, led to a significantly higher number of women reporting subjective improvements after 24 months. Overall, 14.3% of women from the recommendations group and 12.1% from the pessary group switched to another treatment (pessary or surgery) within 24 months. In addition, one out of three women in the recommendations group and one out of four women in the pessary group discontinued the initial treatment without opting for any other POP treatment method. However, for those women who continued the primary treatment, pessary use and surgery showed similar subjective and quality-of-life improvements, although women in the surgery group were significantly older and a had greater impairment in quality of life at baseline, including general health perception, daily role limitations, impact on emotions, and measures of POP severity.

### 4.2. Primary Outcomes

Subjective improvement, measured with the PGI-I scale, showed a significant difference between all three treatment methods (recommendations, pessary, and surgery), with the lowest rate of improvement in the recommendations group and the highest rate in the surgery group. However, women who continued primary pessary treatment reported a high PGI-I success rate of 90.5% at 24 months, which was similar to the PGI-I success rate in the surgery group (89.6%) and consistent with the results of some other studies [14,16]. The proportion of women indicating a subjective improvement with continued pessary use varied from 87.4% at 6 months to 74.4% at 24 months [14,16]. The differences among studies may be attributed to the characteristics of the women involved and the stage of prolapse. In past studies, women opting for pessary treatment were significantly older, had comorbidities, had a lower BMI, and had less advanced stages of prolapse [12,14,16,17,18,19]. Meanwhile, in our study, pessary users were significantly younger than women in the surgery group and predominantly had stage 3 prolapse. It was found that women performing PFMT or PFMT with a structured lifestyle advice program can also achieve a subjective improvement [9,20]. Although in our study, the PGI-I percentage value with continued recommendations increased compared to the baseline, the subjective improvement was significantly lower when compared to the other two treatment methods at 24 months. A woman’s motivation to adhere to a program of PFMT and lifestyle changes is a key factor in its effect. Successful PFMT involves a change in behavior for the woman in order to incorporate exercises and approaches into her daily life. If a woman is not motivated to adhere, the effectiveness of the PFMT and the subjective improvement will be limited. One study in Spain showed that most women, over time, do not perform regular pelvic floor muscle exercises [21].

### 4.3. Secondary Outcomes

Women in the surgery group had a greater quality-of-life impairment at baseline with respect to general health perception, daily role limitations, the impact on emotions, and the POP severity measures when compared with the other two groups. These findings are in line with another study, where women in the surgery group showed greater impairment in role limitations, physical and social limitations, and emotional problems at the baseline when compared with pessary users [22]. It appears that women who experience more symptoms of POP and a greater impairment in quality of life are more likely to choose and undergo surgery.

There was no significant difference in the P-QoL domain scores between the pessary and surgery groups at the 24-month follow-up, while the quality-of-life of women in the recommendations group was significantly worse. The medians of the P-QoL domain scores at 24 months in the prolapse surgery group were 0 in all nine domains, while in the pessary group, this was the case in seven out of the nine domains (except personal relationships and severity measures). These results are consistent with the findings of other studies [12,23]. This systematic review also demonstrated that a pessary can have a similar positive effect on women’s quality of life to surgery and that the improvement in quality of life increased with a longer duration of vaginal pessary use [13]. With regard to the personal relationships domain, the P-QoL scores at the baseline and during the follow-up did not differ significantly among three treatment groups. The reasons could have been the women’s age, comorbidities, and the absence of sexual activity. Similar reasons were expressed in Brazilian research, where no change in the personal relationships domain was found because only one out of three women were sexually active [24]. Vaginal atrophy and postmenopausal status could also exert an impact on women’s sexual life; in our study, significantly more women in the pessary (92.3%) and surgery (96.2%) groups were postmenopausal and had vaginal atrophy. However, in another study where only 16.5% of participants were sexually active, an improvement in sexual symptoms was observed after using a vaginal pessary together with estriol vaginal cream. The authors speculated that sexual symptoms were associated with the usage of cream that could have improved lubrication [9]. 

With regard to adherence to recommendations, the P-QoL scores at 24 months were significantly higher in the recommendations group in most domains, except for the social limitations and personal relationships domains, compared with the other two groups. Similar findings were observed in a study from Thailand, where post-treatment P-QoL scores in the vaginal pessary group were significantly lower than those in the PFMT group. This can be explained by understanding that even when PFMT is performed regularly for a long time, other factors could impact the outcome of the PFMT, such as low baseline pelvic floor muscle strength or a higher BMI [25].

The continuation rate at 24 months was 63.6% in the pessary group and 48% in the recommendations group. These findings are consistent with other studies, where the continuation of pessary treatment varies from 51–63% to 76–78.2% of women within 12 to 24 months [11,13,14,15,26]. Overall, 14.3% of women from the recommendations group and 12.1% from the pessary group switched to another treatment (pessary or surgery) within 24 months, while the rest of the study participants discontinued the initial treatment without opting for any other POP treatment method. The crossover rate from the pessary treatment to surgery was much lower in our study than in other observational cohort studies (23.6%) or randomized controlled trials (54.1%) [14,27]. 

The most common reasons for discontinuation or switching to another treatment method were inadequate symptom relief and a mismatch of expectations in the recommendations group, followed by excessive vaginal discharge, vaginal pain, discomfort, preference for surgery, and pessary expulsion in the pessary group, which are consistent with other studies [6,10,14,19,22,27]. In our study, we did not undertake follow-up visits with the women; this is why we could not encourage them to carry out further pelvic floor muscle training or adhere to lifestyle changes. Since the impact of PFMT on POP comes only when performing it regularly and after a longer period of time, women who do not observe quick effect discontinue or change this treatment method to another one. With regard to pessary use, women may be reluctant to continue pessary therapy if the subjective improvement does not meet their expectations, especially with the knowledge that they can switch to surgery.

### 4.4. Study Strengths and Limitations

We are not aware of another observational prospective study in Lithuania that evaluated global improvement and quality of life after different treatments for POP over a period of 24 months. For outcome measures, a validated Lithuanian version of the P-QoL questionnaire was used. However, our study has several limitations. It was a single-center study. As the objective of this cohort was not to study the non-inferiority of one treatment method versus another, the sample size was chosen pragmatically. Women were assigned to different therapy categories based on their preferences, which we believe could have influenced the results. Randomizing the study participants could have strengthened the study. However, some researchers encountered difficulties during the randomization process because many women strongly preferred one treatment option over the others and did not consent to randomization. Consequently, the randomized controlled trial was prematurely terminated, and a prospective cohort study was conducted [11]. Vaginal pessaries were not provided free of charge, and women who could not afford them could not choose this method of treatment. Information about PFMT was provided and explained but was not individualized, and the PFMT was self-performed. Finally, due to the COVID-19 pandemic, the follow-up visit at 24 months was changed to a phone interview, and this could have had an impact on the results. Despite all the mentioned limitations, this study reflects real-world daily practice. Subjective improvement and quality-of-life changes after different POP treatment methods should be evaluated as relevant patient-reported outcome measures, while the findings of this study could help in the proper counselling of women with POP. The main goal should be an individualized treatment approach that takes into account the patient’s preferences, characteristics of the women, severity of the prolapse, and potential for improvement.

## 5. Conclusions

Vaginal pessaries and surgery showed a clinically important improvement and can be proposed as primary treatment methods for pelvic organ prolapse. Surgery resulted in significantly more women reporting a subjective improvement after 24 months. However, women should be informed that after 24 months, 90.5% of those who continued pessary use reported a subjective improvement compared to 89.6% after surgery. Overall, 12.1% of women who started with pessary treatment switched to surgery, and 24.2% discontinued pessary use without opting for any other POP treatment method because of insufficient symptom relief or side effects. While offering lifestyle changes and PFMT to women with prolapse as options to treat their symptoms is useful, the subjective improvement is lower compared to pessary use or surgery.

Future research involving larger cohorts could offer a more robust analysis of treatment outcomes, potentially uncovering more detailed differences between treatment groups. Furthermore, a more thorough exploration of the reasons for the discontinuation of primary treatment or transition to alternative methods could provide deeper insights into patient satisfaction, treatment efficacy, and potential barriers to adherence.

## Figures and Tables

**Table 1:** Baseline demographic and clinical characteristics of study participants (*n* = 137).

	Recommendations (*n* = 45)	Pessary (*n* = 39)	Surgery (*n* = 53)	*p* Value
Age (median (IQR))	57 (51–66) ^a^	63 (58–68.5) ^b^	70 (61–76) ^ab^	<0.001 *
BMI (kg/m^2^, median (IQR))	27.45 (24.6–32.8)	26.4 (25.14–29.5)	28.95 (24.1–31.62)	0.847 *
Smoking (*n* (%))	6 (13.3)	2 (5.1)	1 (1.9)	0.068 **
Constipation (*n* (%))	14 (31.1)	9 (23.1)	13 (24.5)	0.659 **
Parity (median (IQR))	2 (2–2)	2 (2–3)	2 (2–3)	0.342 *
Vaginal delivery (*n* (%))				
0	1 (2.2)	0 (0)	2 (3.8)	0.376 **
1	10 (22.2)	4 (10.5)	5 (9.6)	
2	26 (57.8)	23 (60.5)	32 (61.5)	
≥3	8 (17.8)	11 (28.9)	13 (25)	
Cesarean section (*n* (%))	4 (8.9)	2 (5.3)	1 (1.9)	0.285 **
Postmenopausal status (*n* (%))	30 (66.7) ^ac^	36 (92.3) ^c^	51 (96.2) ^a^	<0.001 **
Vaginal atrophy (*n* (%))	28 (62.2) ^ac^	35 (89.7) ^c^	47 (88.7) ^a^	<0.001 **
POP-Q stage (*n* (%))				
1	11 (24.4) ^ac^	1 (2.6) ^c^	0 (0) ^a^	<0.001 **
2	25 (55.6) ^d^	6 (15.4) ^d^	1 (1.9) ^d^	
3	9 (20) ^ac^	32 (82.1) ^c^	47 (88.7) ^a^	
4	0 (0) ^a^	0 (0) ^b^	5 (9.4) ^ab^	

IQR—interquartile range, BMI—body mass index, POP-Q—Pelvic Organ Prolapse Quantification. * Kruskal–Wallis test; ** non-parametric chi-squared test. ^a^ significant difference between recommendations and surgery groups. ^b^ significant difference between pessary and surgery groups. ^c^ significant difference between recommendations and pessary groups. ^d^ significant difference between all groups.

**Table 2:** Patient Global Impression of Improvement (PGI-I) rate at 24 months.

	Primary Treatment Methods				Continued Primary Treatment Methods			
	Recommendations (*n* = 35)	Pessary (*n* = 33)	Surgery (*n* = 48)	*p* Value *	Recommendations (*n* = 17)	Pessary (*n* = 21)	Surgery (*n* = 48)	*p* Value *
PGI-I: improvement (*n* (%))	8 (22.9) ^a^	22 (66.7) ^a^	43 (89.6) ^a^	<0.001	6 (35.3) ^bc^	19 (90.5) ^b^	43 (89.6) ^c^	<0.001

* Non-parametric chi-squared test. ^a^ significant difference between all groups. ^b^ significant difference between recommendations and pessary groups. ^c^ significant difference between recommendations and surgery groups.

**Table 3:** Data concerning the primary treatment in the recommendations and pessary groups.

	Recommendations (*n* = 35)	Pessary (*n* = 33)
Reasons for discontinuation or switching to another treatment method (*n* (%))		
Inadequate symptom relief/did not meet expectations	8 (22.9)	3 (9.1)
Excessive vaginal discharge	0 (0)	3 (9.1)
Pessary expulsion	n/a	1 (3.0)
Vaginal pain, discomfort	3 (8.6)	3 (9.1)
No reason	6 (17.1)	0 (0)
Prefer surgery	1 (2.9)	2 (6.0)
Another treatment used (*n* (%))		
Pessary	4 (11.4)	n/a
Prolapse surgery	1 (2.9)	4 (12.1)
Period of primary treatment (median month (IQR))	2 (0–5)	3 (3–3)

n/a: not applicable.

**Table 4:** The Prolapse Quality-of-Life (P-QoL) domain scores in all three groups.

	Baseline				24 Months			
P-QoL Domains	Recommendations (*n* = 35) Median (IQR)	Pessary (*n* = 33) Median (IQR)	Surgery (*n* = 48) Median (IQR)	*p* Value *	Recommendations (*n* = 17) Median (IQR)	Pessary (*n* = 21) Median (IQR)	Surgery (*n* = 48) Median (IQR)	*p* Value *
General health perception	50 (37.5–50) ^a^	50 (50–50)	50 (50–75) ^a^	0.008	50 (25–50) ^ab^	0 (0–25) ^b^	0 (0–25) ^a^	<0.001
Prolapse impact	66.67 (33.33–100)	66.67 (66.67–100)	66.67 (66.67–100)	0.059	33.33 (33.33–66.67) ^ab^	0 (0–33.33) ^b^	0 (0–0) ^a^	<0.001
Role limitations	33.33 (8.33–58.33) ^a^	33.33 (0–66.67) ^c^	66.67 (33.33–83.33) ^ac^	0.005	0 (0–33.33)^a^	0 (0–0)	0 (0–0) ^a^	0.016
Physical limitations	50 (16.67–83.33)	50 (16.67–66.67)	66.67 (33.33–100)	0.082	16.67 (0–50) ^ab^	0 (0–16.67) ^b^	0 (0–0) ^a^	0.003
Social limitations	11.11 (0–38.89)	0 (0–33.33)	22.22 (0–61.11)	0.068	0 (0–22.22)	0 (0–0)	0 (0–0)	0.149
Personal relationships	33.3 (0–66.6)	16.6 (0–33.33)	50 (33.3–83.3)	0.142	50 (0–100)	33.33 (0–66.66)	0 (0–0)	0.110
Emotional disturbances	33.33 (11.11–77.78) ^a^	33.33 (22.22–66.67) ^c^	66.67 (33.33–88.89) ^ac^	0.018	0 (0–33.33) ^ab^	0 (0–0) ^b^	0 (0–0) ^a^	0.007
Sleep or energy disturbances	33.33 (0–50)	16.67 (0–33.33)	33.33 (16.67–50)	0.071	33.33 (0–66.67) ^ab^	0 (0–16.67) ^b^	0 (0–0) ^a^	<0.001
Severity measures score	25 (16.67–33.33) ^a^	25 (16.67–41.67) ^c^	37.5 (20.83–66.67) ^ac^	0.009	16.67 (0–41.67) ^a^	8.33 (0–25)	0 (0–8.33) ^a^	0.007

* Kruskal–Wallis test. ^a^ statistically significant difference between recommendations and surgery groups. ^b^ statistically significant difference between recommendations and pessary groups. ^c^ statistically significant difference between pessary and surgery groups.

## Data Availability

Data are available from the corresponding author upon reasonable request.
